# Synthetic Cannabinoids in Sierra Leone: Understanding the Use of ‘Kush’ Among Youths and Its Socioeconomic Impact in Sierra Leone and Sub‐Region

**DOI:** 10.1002/puh2.70031

**Published:** 2025-02-06

**Authors:** Michael Lahai, Ahmed Vandy, Alvin Turay, Marie Kolipha‐Kamara, Eugene Conteh

**Affiliations:** ^1^ Faculty of Pharmaceutical Sciences, College of Medicine and Allied Health Sciences University of Sierra Leone Freetown Sierra Leone

**Keywords:** illicit drugs, Kush, substance use disorder, synthetic cannabinoids, youth mental health

## Abstract

Sierra Leone and neighbouring countries are prone to the proliferation of illicit drugs due to porous borders, weak regulatory frameworks, and the activities of transnational criminal syndicates. Among the emerging drug threats in the region is the synthetic cannabinoid known as ‘Kush’, which has gained immense popularity. The use of this drug has surged in recent years, particularly among Sierra Leonean youths, leading to the declaration of a national state of emergency by the government. The Ministry of Health and Sanitation has been tasked with establishing a National Task Force on drugs and substance abuse to identify treatment and mitigation measures to combat Kush addiction among affected individuals. This commentary highlights the current situation of Kush abuse among youths, its usage patterns and socioeconomic implications for Sierra Leone and the surrounding countries with key recommendations that will inform strategies for prevention, treatment and regulation.

## 1 Introduction

Sierra Leone and its neighbouring countries in West Africa persistently face challenges related to porous borders, extensive coastlines and limited law enforcement resources in regulating the movement of goods and people across their borders. The weak drug legislation, coupled with understaffed and under‐resourced regulatory and customs agencies are vulnerabilities exploited by drug traffickers, contributing to the influx of illegal drugs in the region [1]. Among the emerging drug threats in sub‐Saharan Africa is the synthetic cannabinoid known as ‘Kush’, in Sierra Leone and a similar addictive substance called ‘Colorado’ in Nigeria. These addictive substances have gained widespread popularity in West Africa with an increase in addiction cases among youths [2, 3]. In 2023, Sierra Leone served as the epicentre of the Kush crisis, with its usage exceeding that of many other countries in the West Africa region, including Liberia, Guinea, the Gambia, Nigeria and Côte d'Ivoire [2]. A study in Kambia District, Sierra Leone that was published in 2023 found an 86.7% prevalence of substance use among commercial motorcyclists. ‘Kush’ and marijuana were identified as the most abused substances, with 29.1% and 22.0% usage rates, respectively [4]. Other substances which were also used by the participants were Alcohol, Tramadol and Relief tablets. Another study conducted at the Sierra Leone Psychiatric Teaching Hospital examining psychoactive substance use found that 59% of the 719 admitted patients were Kush users [5]. Although some of these patients also used other substances in addition to the Kush, the percentage of single substance users who were on Kush alone was found to be 58% (100/172).

The Ministry of Health and Sanitation (MOHS) and Human Rights Commission of Sierra Leone in March 2024 reported the mass burial of 32 unidentified youths (25 men and 7 women) who were alleged victims of Kush intoxication. Additionally, numerous deaths have been linked to this drug, raising serious concerns and highlighting the urgent need for intervention. Currently, there is no official data on the number of affected victims and deaths, but reports from the British Broadcasting Corporation revealed that hundreds of youths in the country have died due to organ failure caused by the drug. The number of cases admitted for rehabilitation at the Sierra Leone Psychiatric Hospital between 2022 and 2023 amounts to 1865, a 4000% increase in hospital admissions [6].

Kush's affordability and high profit margins for dealers have made it a lucrative business for drug traffickers, leading to a significant increase in its consumption among the youth [3]. Porous borders, socioeconomic disparities and recovery from outbreaks and post‐war trauma could also heighten the vulnerability of youths to the use of these illicit drugs [7, 8, 9]. High unemployment rates and the aftermath of the Ebola epidemic and COVID‐19 pandemic could also strain the mental health status of youths in the West African region.

Sierra Leone faces a treatment gap for mental illnesses, with only one psychiatric hospital serving the entire nation [10]. These mental health challenges have further created a vulnerable environment, increasing the risk of substance abuse and involvement in illicit drug use (including Kush consumption) among youths. Transnational criminal syndicates have capitalised on these vulnerabilities, with further exacerbation in the influx of illicit substances and their associated societal consequences. The surge in Kush consumption has led to increased addiction and health issues, thereby straining the already limited healthcare system and hindering workforce productivity and economic growth, while contributing to social disintegration and stigmatisation. In response to the Kush crisis, on 5 March 2024, Sierra Leone officially declared a public health emergency against drugs and substance abuse. Drugs and substance abuse public health emergency was identified as an epidemic and a significant national peril that was described by the President as a ‘death trap posing existential crisis’ [11]. The established National Task Force of the MOHS has been assigned with the responsibility of combating drugs and substance abuse, with a focus on community involvement and engagement, prevention of potential new cases of drug abuse, treatment of affected individuals, and the cooperation of regulatory agencies and law enforcement agencies to track down drug traffickers [6].

## 2 Kush: Composition, Variants and Effects

Kush is a relatively new psychoactive drug, believed to be a synthetic cannabinoid by the Sierra Leonean Drug Law Enforcement Agency [3]. Its chemical composition is constantly evolving, which makes it a complex and challenging substance to study. The Kush in Sierra Leone has been described as a polydrug mixture that is known to contain a variety of chemicals which are prepared and sprayed onto dry leaves for smoking. The gap in laboratory studies makes it unclear to provide an evidence‐based classification of the synthetic drug. However, some sources have revealed the presence of cannabis‐like substances and other drug substances like fentanyl, tramadol, formaldehyde and possibly ground‐down human bones, mixed by local criminal gangs [12]. The texture and content are also known to be different by location and by producer. A recent Fourier transform infrared spectroscopy testing of retail Kush sold in Sierra Leone and Guinea‐Bissau detected the presence of synthetic cannabinoids and classified Kush into three categories based on the presence of the synthetic cannabinoid component: Type A contains synthetic cannabinoid as the active ingredient, Type B contains synthetic cannabinoid as the primary active ingredient and nitazene as the secondary active ingredient, and Type C which contains nitazene as the primary active ingredient [13]. About 72% of the samples found in Sierra Leone were made of Type C, 17% of Type A and 11% of Type B. Nitazenes were found to be present in 83% and 55% of samples tested in respective countries (Sierra Leone and Guinea‐Bissau). No fentanyl or phencyclidine (PCP) was, however, detected in the Kush samples tested. However, the study has yet to provide an advanced confirmatory test to conclude the class of synthetic cannabinoids used for preparing Kush in these countries.

The drug is believed to have international sources, with cannabis and related leaves widely grown in Sierra Leone and fentanyl originating in clandestine laboratories overseas. It has recently been discovered that human bones are added to the concoction to enhance their potency, possibly because of the sulphur content in the bones. There is no scientific indication to support this claim of using human remains in the drug concoction; however, credible sources have reported that dealers suspected to be engaged in the production of the drug are digging up skeletons and retrieving ground‐up human bones from cemeteries in Freetown [6, 14]. Other scholarly articles have revealed that Kush is prepared from manufactured chemicals that are usually sprayed on plant material to be smoked [15]. While intended to replicate the effects of tetrahydrocannabinol, it is more potent and addictive and causes a wide spectrum of side effects compared to natural cannabis [15].

The effects of Kush differ based on the user, its content, and the amount ingested. According to a study conducted in Sierra Leone, the use of different Kush variants depends on the desired effect [14]. ‘Boss Kush’ is reportedly used to enhance productivity and alertness, while some individuals prefer the potent high of ‘Jagaban Kush’. In contrast, ‘Gentle Kush’ is chosen by users seeking relaxation and improved sleep. However, certain strains, such as ‘Tramadol Kush’, are associated with serious health risks such as hematemesis. The Cannabis content can induce euphoria, relaxation, and an altered state of consciousness, whereas the inclusion of potent opioids like fentanyl and tramadol can cause severe sedation, confusion and respiratory depression [16, 17]. Kush users, predominantly males aged 18–25, have exhibited a ‘zombie‐like’ gait, collapsing unexpectedly, and exhibiting symptoms of cognitive impairment and psychosis [18]. Psychosis, delirium and agitation are the common psychiatric symptoms which have been associated with Kush and related synthetic cannabinoid toxicity due to its binding affinity to the cannabinoid receptors (CB_1_ and CB_2_) producing tetrahydrocannabinol‐like neuropsychiatric manifestations [19, 20, 21]. Long‐term use of the drug has been associated with large necrotizing wounds on users’ feet, liver, kidney and respiratory problems and ultimately death [22].

## 3 The Need to Improve Mental Healthcare Landscape in Sierra Leone

Sierra Leone has endured a series of traumatic events that may have profoundly affected the mental health of its citizens. The country has grappled with a decade‐long civil war, devastating pandemics, natural disasters like mudslides and persistent economic instability characterized by high unemployment rates [23, 24]. These adversities have led to the deterioration of mental health, which may have fuelled the increase in substance use. Many Sierra Leoneans, especially youth, are turning to Kush as a form of self‐medication to cope with economic hardship and psychological distress [14]. Negative attitudes towards these victims with mental illness, often stemming from ignorance, result in neglect, abuse and violation of their dignity and basic human rights [25].

The mental healthcare landscape is in a critical state and dear need, with a treatment gap so vast that only 2% of the estimated 102,000 individuals suffering from severe mental illness receive medical care [10]. Limited access to formal mental health services has led many individuals to seek alternative treatments, such as traditional healers, before accessing the formal healthcare system [24]. This reflects the significant investment in and reliance on traditional and informal methods of addressing mental health issues within the country. The lack of mental health resources and the stigma associated with seeking professional help have also created a vicious cycle, where those in need of support are often unable to access the necessary care. The country's sole inpatient psychiatric facility, the Sierra Leone Psychiatric Hospital, is plagued by underfunding, insufficient human resources and a lack of basic amenities, further exacerbating the challenges of providing adequate care [24]. With only 2 psychiatrists, 2 clinical psychologists, 19 mental health nurses with 1 Pharmacist serving a population of over 7 million [26], the demand for mental health services far exceeds the available resources [26]. Certainly, the ratio of psychiatrists, nurses, and pharmacists does not meet the required ratio of specialist health professionals to patients as described by WHO [10]. This trend underscores the urgent need for improved mental healthcare services within the country.

An effective mental health response involves facilitating the reintegration of service users into society and enabling them to make meaningful contributions to their communities [25]. This approach not only restores their dignity but also transforms societal perceptions of mental illness and the journey to recovery. WHO and partners are collaborating to strengthen mental health services within the MOHS in Sierra Leone. This includes providing technical support for reviewing the ‘Lunacy Act’ of 1902, revising the Mental Health Policy and Strategic Plan and training healthcare workers [26]. Additionally, engaging with communities, religious leaders and civil society organizations to destigmatize mental health issues and promote early intervention and preventive measures can significantly improve access to mental health services. Addressing the underlying socioeconomic factors, such as unemployment and economic instability, can also help alleviate the psychological distress experienced by the population.

## 4 Recommendations and the Way Forward

To address the Kush crisis in Sierra Leone and the sub‐Saharan Africa region, a multifaceted approach is required. This includes incorporating the following key elements:

- Strengthening border management and control operations, enhancing regional and international cooperation to disrupt cross‐border drug trafficking and implementing robust monitoring and enforcement mechanisms are crucial steps in curbing the influx of illicit substances in the West African region.
- Investing in mental health infrastructure, training mental health specialists in all professional cadres (medical doctors, pharmacists and nurses) and increasing the availability of treatment and rehabilitation services.
- Addressing the underlying socioeconomic challenges is essential for tackling the root causes of substance abuse. Centres should be established to provide not only medical treatment but also social and vocational rehabilitation services to meet the diverse needs of Kush users and other individuals with substance abuse issues.
- Community‐based prevention programmes, educational campaigns and targeted interventions aimed at identifying vulnerable populations can play a vital role in mitigating the widespread use and adverse effects of Kush.
- Given Kush's complex addictive nature, a harm reduction approach may be more effective than focusing solely on immediate cessation. Individuals addicted to Kush and caregivers should receive non‐judgemental education on its risks, with addiction treated as a health issue rather than a crime.
- Fostering collaboration between law enforcement agencies, healthcare providers and community organisations to develop holistic interventions.
- Reviewing regulatory frameworks and policies to control the production, distribution and consumption of narcotics and illicit drugs.
- Conducting further research to better understand the long‐term effects of Kush and develop targeted harm reduction strategies.
- All these programmes must be implemented with a strong background of quality improvement and process assessment.
- There is a need for well‐equipped laboratories where the full chemical profile of the drug and other related substances can be determined.
- There is a need for studies investigating the prevalence of Kush use in a large population.

## 5 Conclusion

The Kush crisis in Sierra Leone and the West African region has far‐reaching implications for public health, economic development and social stability in the region. The legacies of trauma, the lack of adequate healthcare infrastructure and the reliance on traditional healing practices in rural and peri‐urban communities have all contributed to the deterioration of the country's mental health landscape and increased vulnerability of our youths to illicit drug use. Addressing this complex challenge requires a collaborative, multifaceted approach that tackles the drug supply sources within the country, border strengthening and developing programmes aimed at addressing and improving the underlying factors responsible for mental health issues among youths. By implementing a comprehensive strategy, Sierra Leone and its neighbours can work towards mitigating the devastating impacts of Kush and fostering a more resilient and healthier future for their communities.

## Author Contributions

Michael Lahai established the need for the commentary. Michael Lahai, Alvin Turay and Ahmed Vandy conceptualise the commentary idea. Alvin Turay, Ahmed Vandy and Michael Lahai provided the original draft of the manuscript. Michael Lahai, Ahmed Vandy, Eugene Conteh and Marie Kolipha‐Kamara manuscript review. All authors have read the content of this article and approved its submission for publication.

## Ethics Statement

The authors have nothing to report.

## Consent

The authors have nothing to report.

## Conflicts of Interest

The authors declare no conflicts of interest.

## Data Availability

All required information has been provided in this manuscript.
